# Impact of fetal maceration grade on risk of maternal disseminated intravascular coagulation after intrauterine fetal death – A retrospective cohort study

**DOI:** 10.1038/s41598-018-30687-0

**Published:** 2018-08-24

**Authors:** Dana A. Muin, Helmuth Haslacher, Vanessa Koller, Herbert Kiss, Anke Scharrer, Alex Farr

**Affiliations:** 1 Department of Obstetrics and Gynecology, Division of Fetomaternal Medicine, Medical University of Vienna, 1090 Vienna, Austria; 2 Tommy’s Stillbirth Research Center, Faculty of Biology, Medicine and Health, The University of Manchester, Manchester, M13 9WL United Kingdom; 3 Department of Laboratory Medicine, Medical University of Vienna, 1090 Vienna, Austria; 4 Clinical Institute for Pathology, Medical University of Vienna, 1090 Vienna, Austria

## Abstract

Disseminated intravascular coagulation (DIC) is a life-threatening event that is the endpoint of a pathologically activated cascade leading to excessive consumption of platelets culminating in bleeding. Several diseases are known to be associated with DIC, some of which may also occur during pregnancy or the puerperium. One of the potential risk factors that have been considered as a potential trigger for DIC is the retention of a highly macerated fetus after intrauterine fetal death (IUFD). However, sparse evidence exists on its clinical implication on hemostasis parameters. In this retrospective single-center study, we investigated the role of fetal maceration grades 0-III on the risk of DIC in 91 women following IUFD between gestational weeks (+days) 22 + 0 and 41 + 6 between 2003 and 2017. We calculated the Erez DIC-score after consideration of maternal platelet count (PC), prothrombin time (PT) and fibrinogen (Fib) and correlated the findings with fetal maceration grade. Mean (±SD) age of women was 32.1 ± 6.7 years. Neither maternal hemostasis parameters (PC, PT, Fib), nor the Erez score showed a statistically significant difference between maceration grades 0-III with median values of 1 for all four grades (maceration grade I: range 0 to 27; I: 0 to 51; II: 0 to 52; III: 0 to 39). We therefore conclude, that the pathophysiology of DIC in women after singleton IUFD is unrelated to the degree of fetal maceration.

## Introduction

Disseminated intravascular coagulation (DIC) is a clinicopathological syndrome characterized by the formation of fibrin clots with concomitant consumption of platelets and coagulation factors that leads to organ failure and contributes to a high mortality if left untreated^1–3^. In pregnancy, there is a physiological increase in coagulation factors I (fibrinogen), VII, VIII, IX and X, while other plasma factors and platelets remain stable^4^, all serving the maintenance of the utero-placental interface with the aim of preventing life-threatening hemorrhage after delivery^5^. Yet, upon destruction of maternal tissue and release of collagen and tissue components from the feto-maternal complex into maternal circulation, coagulation may be activated by thromboplastin via the extrinsic pathway. This causes tissue factors to be released, building a complex with factor VII and activating factors IX and X, finally culminating in DIC. Obstetrical conditions associated with DIC include placental abruption due to a large amount of released collagen^6–9^, amniotic fluid embolism with circulating mucin cells causing rapid defibrinolysis in maternal circulation^10,11^, maternal septic shock with hematogenous spread of exotoxins, endotoxins and tissue damage accompanied by acidosis^12–16^, as well as preeclampsia and HELLP syndrome due to hypercoagulation and endothelial injury. In addition, the consumption of coagulation factors can lead to massive obstetric hemorrhage, and acute fatty liver during pregnancy may cause tissue factors and anti-thrombin to enter the blood stream^3,17^.

Fetal maceration takes place upon intrauterine fetal death (IUFD) and is a process characterized by enzymatic autolysis of cells and degeneration of connective tissue leading to skin discoloration, desquamation with formation of bullae and eventually skin peeling, as well as edema of the outer and inner organs with turbid effusions inside the fetal body and amniotic cavity^18^. After a week, the skull bones loosen their conjunction and start to overlap as recognized as the “Spalding sign” on ultrasound. Retention of a dead fetus in the uterine cavity for over 8 days, reaching maceration at stage III^19,20^, was thought to lead to maternal DIC, a condition called “fetal demise syndrome” by Romero *et al*. in 1985^21^. In fact, the role of fetal maceration as a trigger for DIC has been first proposed in 1901 by De Lee, who described “temporary hemophilia” in a woman who had developed a bleeding disorder after delivery of a severely macerated fetus^22^. Fifty years later, and in a series of subsequent observations of hypofibrinogenemia in women after IUFD, Weiner *et al*. proposed three potential mechanisms for this pathology: decreased hepatic fibrinogen production, increased fibrinolytic activity and disseminated intravascular coagulation^23,24^. Whilst the first pathomechanism was dismissed soon after, the latter has been proposed to be linked to the release of tissue thromboplastin from the fetoplacental unit and thus activation of the extrinsic pathway of the coagulation cascade. More recent evidence shows that women after IUFD are found to have increased *in-vivo* thrombin generation and platelet activation when compared to healthy mothers^25^. Furthermore, their amniotic fluid contains higher levels of tissue factor concentrations indicating higher thrombin generation.

Despite anecdotal case-reports on establishment of DIC in women during or after prolonged retention of a dead fetus, there is a lack of evidence to verify this association. We therefore designed this retrospective cohort study to prove the hypothesis, whether higher grades of fetal maceration, as denominator for prolonged fetal retention, elicit hematological changes in the maternal system and therewith increase the risk of DIC in this population after IUFD. We calculated the Erez score^26^ in all eligible women from our institution between 2003 and 2017 and correlated these results with the fetal maceration grades 0 to III, as obtained from the pathology reports following fetal post-mortem autopsy.

## Results

### Baseline characteristics

A total of 91 women were included in this study whose baseline and fetal characteristics are shown in Table 1. Mean (±SD) maternal age at time of delivery was 32.1 ± 6.7 years; 44 (48.4%) women were Caucasian, 20 (22.0%) were Eastern European, 15 (16.5%) were Turkish, 3 (3.3%) were African, Iranian or Middle Eastern origin, respectively, 2 (2.2%) women were Indian and 1 (1.1%) was US-American; 19 (20.9%) women were smokers and 1 (1.1%) reported alcohol consumption during pregnancy. No illicit drug consumption was reported in this cohort.

**Table 1:** Maternal and fetal baseline characteristics.

		Variable	Number of subjects	Range		Median	Mean	Std. Deviation	Std. Error of Mean
				Minimum	Maximum				
Maternal Characteristics		Age (years)	91	18	45	32	32	6.7	0.7
		BMI (kg/m^2^)	71	19	43	26	26	5.2	0.62
		Gravida	91	1	11	2	2.5	1.9	0.2
		Para	91	0	9	1	1.2	1.5	0.16
		Previous pregnancies (n)	91	0	10	1	1.5	1.9	0.2
		Previous live births (n)	91	0	9	1	1.1	1.5	0.15
		Previous stillbirths (n)	91	0	2	0	0.088	0.32	0.034
		Previous miscarriages (n)	91	0	3	0	0.23	0.56	0.059
		Previous terminations (n)	91	0	2	0	0.077	0.34	0.036
		Gestational weeks	91	22	41	30	31	6.1	0.63
		Gestational days	91	0	6	3	2.9	1.9	0.2
		Blood loss (ml)	89	30	1500	200	257	250	27
Fetal Characteristics	Total	Weight (g)	91	104	4450	971	1456	1057	111
		Length (cm)		20	55	37	39	9.2	0.98
		Head circumference (cm)		22	36	32	30	4.3	0.75
	Male	Weight (g)	48	104	4450	794	1438	1142	165
		Length (cm)		20	55	36	38	9.82	1.45
		Head circumference (cm)		22	36	33	31	4.71	1.05
	Female	Weight (g)	43	237.0	3800.0	1300.0	1477.0	965.0	147
		Length (cm)		22.0	55.0	40.0	39.0	8.68	1.32
		Head circumference (cm)		22.0	35.0	30.0	30.0	3.53	0.98

The diagnosis of IUFD in 48 (52.7%) male and 43 (47.3%) female fetuses was made between 22 + 0 and 41 + 6 gestational weeks at a mean of 31 + 3 ± 6 + 2 weeks. Causes of IUFD according to the Tulip classification were congenital fetal malformations in 15 (16.5%) cases, placental pathologies in 38 (41.8%) cases, cord pathologies in 11 (12.1%) cases, infection in 8 (8.8%) cases and maternal or fetal disease in 7 (7.7%) cases. Causes of IUFD were unknown despite thorough investigation in 11 (12.1%) cases and unknown due to missing information in 1 (1.1%) case.

The mode of delivery was *per vaginam* from cephalic presentation in 76 (83.5%) cases and from breech position in 4 (4.4%) cases. Instrumental delivery was performed in 2 (2.2%) cases and caesarean section was carried out 9 (9.9%) times. A total of 50 (54.9%) women required no analgesia during labor, whereas 28 (30.8%) received epidural anesthesia, while 6 (6.6%) were delivered under spinal and general anesthesia, respectively. 1 (1.1%) woman requested oral analgesia only.

As per autopsy report, 13 (14.3%) fetuses had a maceration grade of 0, 13 (14.3%) had a maceration grade of I, 31 (34.1%) had a maceration grade of II and 34 (37.4%) fetuses were found to have a maceration grade of III. No significant difference between fetal sex and maceration grade was observed (*p* = 0.99).

### Blood loss and disseminated intravascular coagulation

In the study cohort, mean blood loss was 257 ± 250 ml. Minor PPH occurred in 4 (4.4%) women with a mean blood loss of 825.0 ± 50 ml. Major PPH occurred in 3 (3.3%) women with a mean blood loss of 1166.7 ± 288.7 ml.

With regards to the assessment of DIC, an Erez score ≥26 was noted in 6 (6.6%) women ranging from 26 to 52 points, with a mean of 36.8 ± 12.4 (s. Supplementary Table 1).

In women with a positive Erez score, mean PC was 68.2 ± 30.0 G/L, mean PT was 3.4 ± 8.1 sec and mean Fib was 2.2 ± 0.7 g/dl, resulting in a median blood loss of 500 ml (150 to 1000 ml) at the time of delivery or during the early postpartum period. In women with a negative Erez score, mean PC was 227.8 ± 63.1 G/L, mean PT was −4.5 ± 1.3 sec and mean Fib was 5.0 ± 1.1 g/dl, yielding a statistically significant less mean blood loss of 244.4 ± 240.6 ml (*p* = 0.01).

### Influence of fetal maceration grade on hemostasis

**Figure 1:**
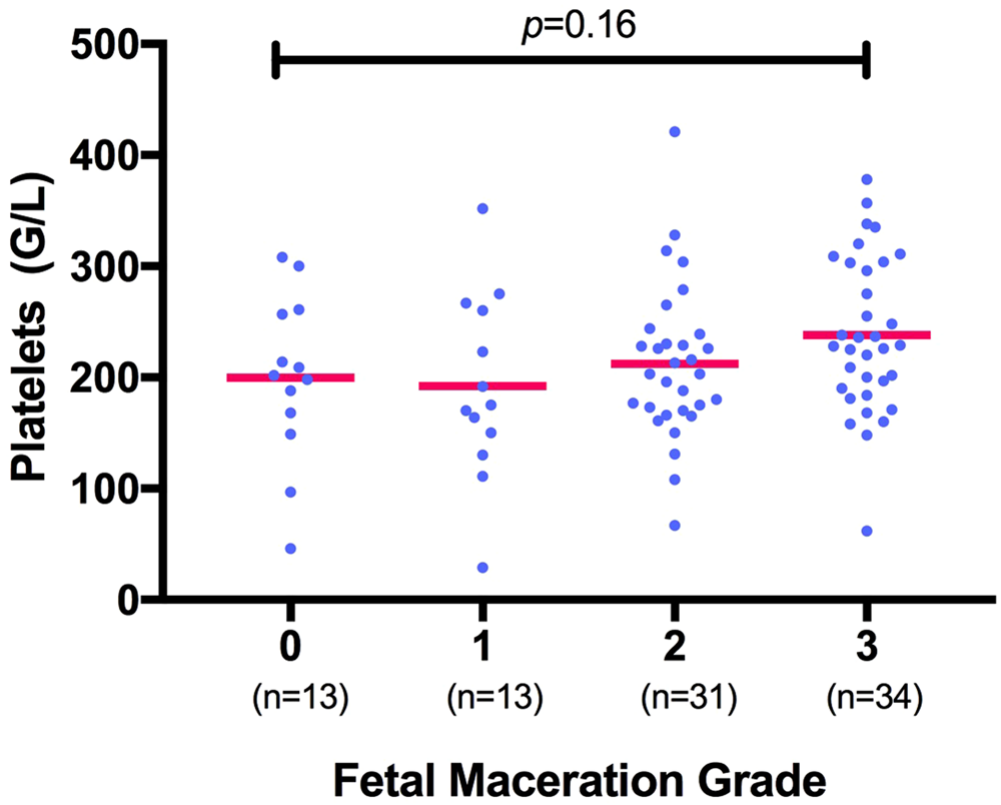
Measurement of platelet count (in G/L) shows no significant correlation with fetal maceration grades 0-III as assessed by Kruskal-Wallis test for non-parametric distribution (n = 91; *p* = 0.16; r^2^ = 0.06).

**Figure 2:**
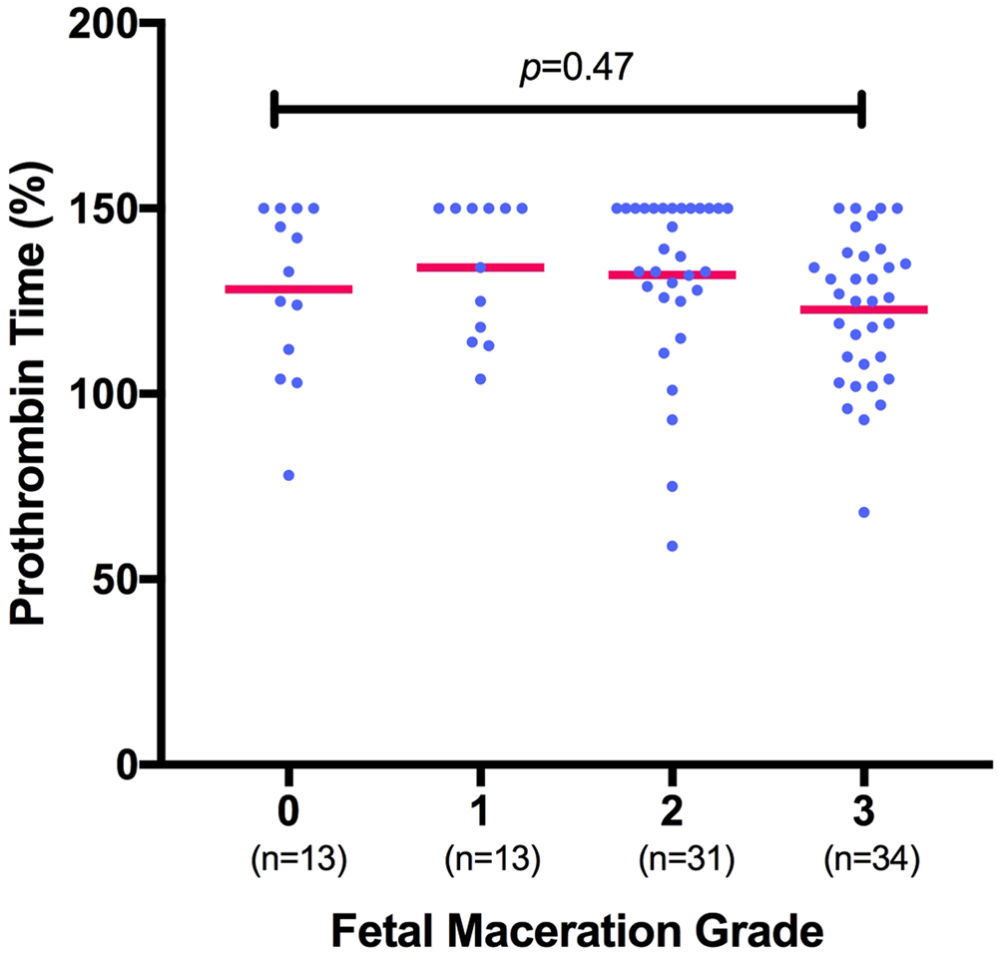
Measurement of prothrombin time (in percentages) shows no significant correlation with fetal maceration grades 0-III as assessed by Kruskal-Wallis test (n = 91; *p* = 0.47; r^2^ = 0.03). One outlier (PT 25%) was excluded from the group with maceration grade I for graphic purpose, yet not from statistical analysis.

**Figure 3:**
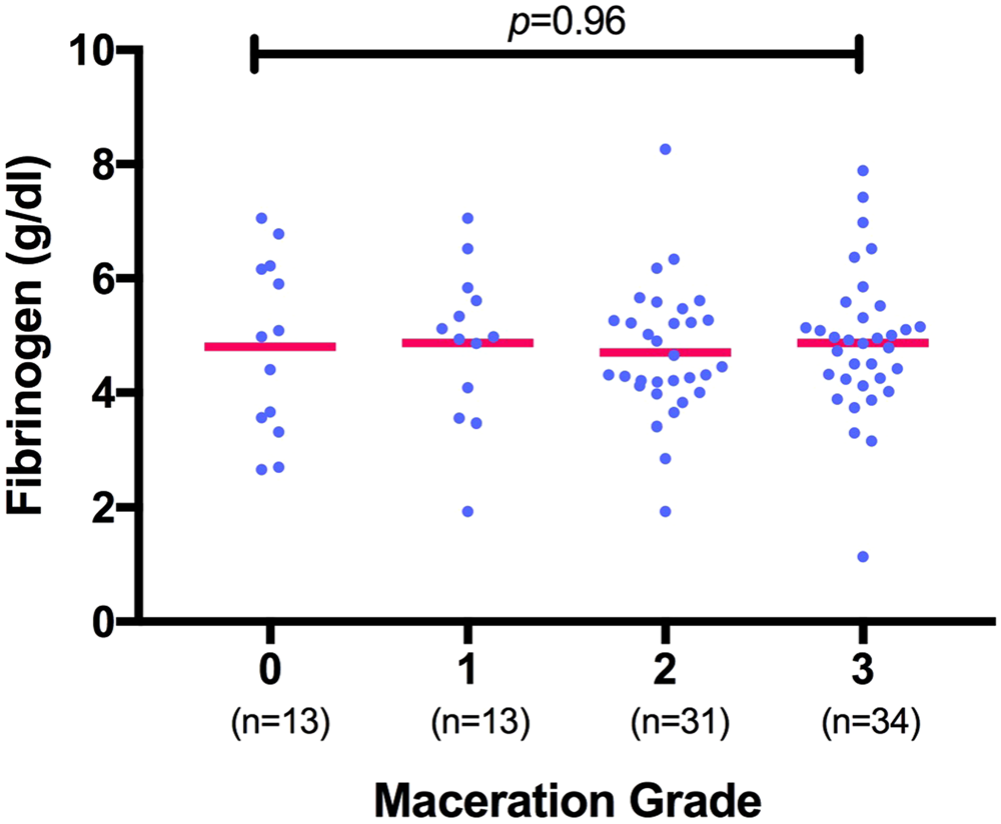
Measurement of fibrinogen (in g/dl) shows no correlation with fetal maceration grades 0-III as assessed by Kruskal-Wallis test (n = 91; *p* = 0.96; r^2^ = 0.04).

Maternal hemostasis parameters (PC, PT, Fib) showed no statistically significant correlation with fetal maceration grades 0-III (n = 91; PC *p* = 0.16; r^2^ = 0.06; PT *p* = 0.47; r^2^ = 0.03; Fib *p* = 0.96; r^2^ = 0.04; s. Figs 1–3).

**Figure 4:**
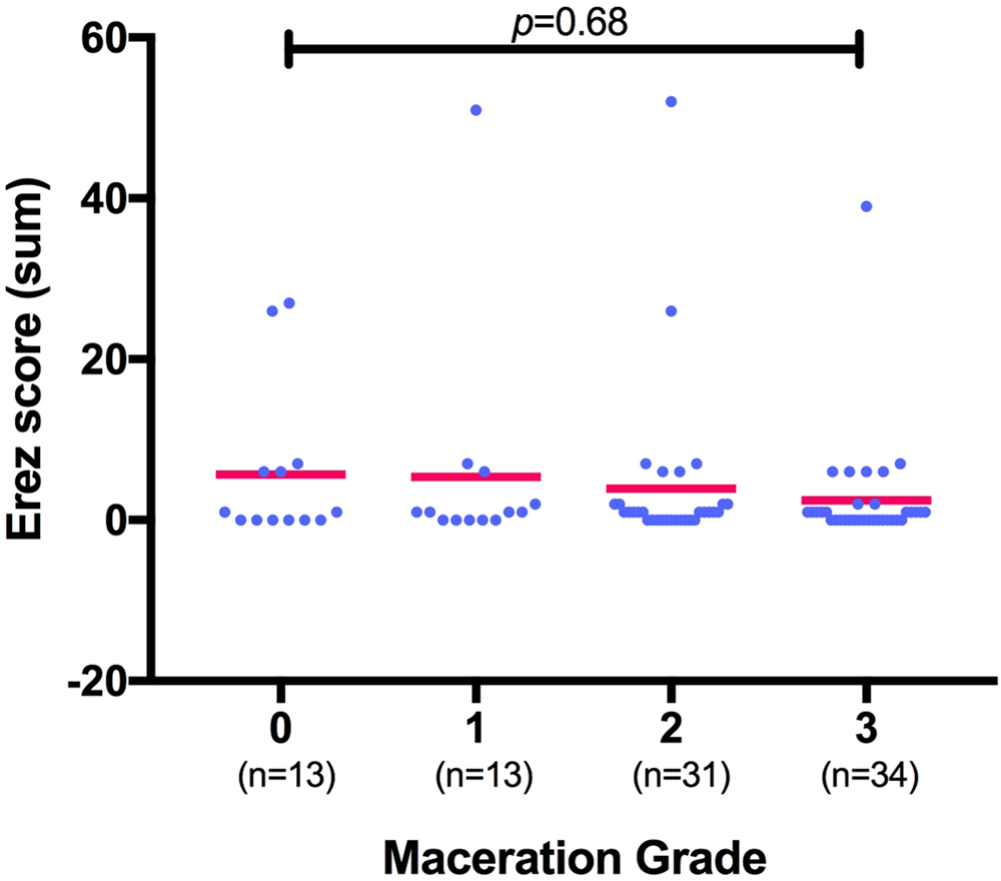
Calculation of the Erez score (as the total sum of PC, PT and Fib) shows no significant difference among fetal maceration grades 0-III as assessed by Kruskal-Wallis test (n = 91; *p* = 0.68; r^2^ = 0.02).

**Figure 5:**
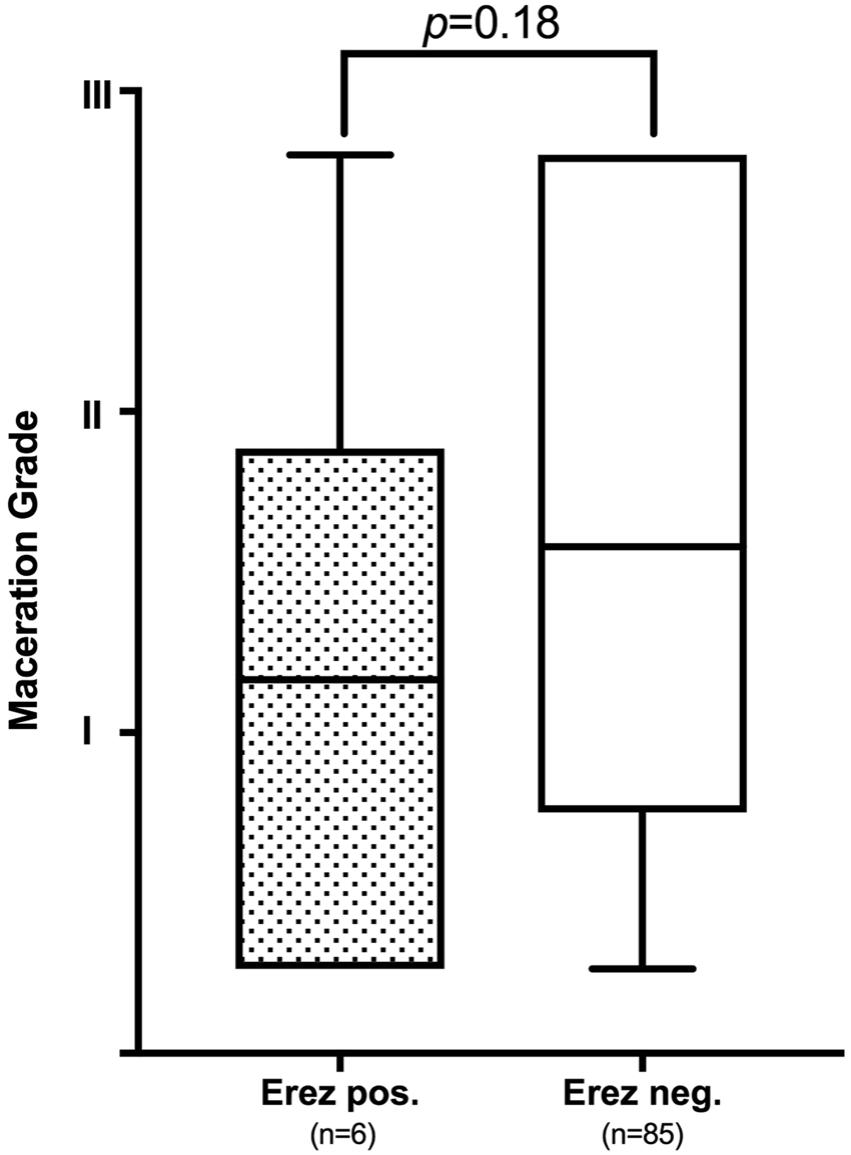
Relationship between Erez score (positive ≥ 26 points; negative ≤ 25 points) and fetal maceration grade 0-III shows no significant correlation by non-parametric Mann-Whitney U test (*p* = 0.18).

Women whose fetus showed a maceration grade of **0** had a mean PC of 200 ± 74.7 G/L, mean PT of 128.2 ± 23.14% and mean Fib of 4.8 ± 1.5 g/dl. Women whose fetus showed a maceration grade of **I** had a mean PC of 192 ± 83.7 G/L, mean PT of 125.6 ± 34.86% and mean Fib of 4.9 ± 1.4 g/dl. Women with a grade **II** macerated fetus had a mean PC of 212 ± 69.1 G/L, mean PT of 132.1 ± 23.35% and mean Fib of 4.7 ± 1.2 g/dl. Women with a grade **III** macerated fetus had a mean PC of 238 ± 69.5 G/L, mean PT of 122.7 ± 19.81% and mean Fib of 4.9 ± 1.3 g/dl. Overall, there was no significant difference in the total numerical Erez calculation among all maceration grades (*p* = 0.68; r^2^ = 0.02; s. Fig. 4): In detail, the Kruskal-Wallis test for comparison of all four maceration grades showed a median value of 1 for maceration grade 0 (range 0–27), maceration grade I (range 0–51), maceration grade II (range 0–52) and maceration grade III (range 0–39). Likewise, the categorization of the Erez sum into a “positive” (≥26 points) versus “negative” (≤25 points) score showed no statistically significant difference between fetal maceration grades 0-III (*p* = 0.18; s. Fig. 5). No statistically significant difference was found in hemostasis parameters with regards to cause of fetal death in-between groups (PC *p* = 0.34; PT *p* = 0.65; Fib *p* = 0.47).

## Discussion

In this study our aim was to explore the impact of fetal maceration grade on the risk of maternal coagulopathy after singleton IUFD, as expressed by pathophysiological changes in biochemical markers and the clinical picture of the patient. We therefore retrieved the hemostasis parameters of all eligible subjects from the last 15 years at our department, calculated their Erez score and correlated the test results with the reported maceration grade of the intrauterine demised fetus. Our main finding was that there is no correlation between fetal maceration grade 0 to III and risk of DIC in women after singleton IUFD. This finding rejects the common hypothesis that there is a clinical correlation between a retained fetal corpse and a higher prevalence of consumptive coagulopathy in pregnant women grounded on the paradigm that fetal tissue factors released into maternal blood stream trigger the activation of consumptive processes^25^.

Despite the fact that several medical textbooks still refer to the “fetal demise syndrome” or “dead fetus syndrome”^3,17,18^, there is only a paucity of data to support this, thus being dismissed by some authors^27–29^. After all, the presence of a “vanishing twin”, which refers to the disappearance of one sac or embryo from a primary twin pregnancy during the first trimester, due to either chromosomal abnormalities and placental dysfunction^30^, may occur in 10.4% to 24% of pregnancies after assisted reproductive techniques^31,32^. Following the justification that early embryonic loss in a twin pregnancy might still be related with a release of tissue factors to guide the autolytic process of the fetus, one might assume that women after early fetal loss are prone to laboratory changes as described by Erez *et al*.^25^. However, the vanishing of a deceased twin might conclude well before the maceration process takes place. Indeed, to date, no figures have been published on the incidence of coagulopathy in women following vanishing twin syndrome^33^. On the contrary, maceration, or even mummification in terms of a “fetus papyraceus” are a common feature in cases where selective fetal reduction (feticide) had been performed based upon the prenatal diagnosis of severe fetal malformations affecting one of the multiples, or following higher order multiple pregnancies after assisted reproduction^34^. Contrasting many retrospective cohort studies on the prevalence of maternal coagulopathy within the frame of the “fetal demise syndrome” following selective termination in a twin pregnancy^35–37^, only one case-report has been published so far on a 23-year-old woman who had developed DIC following selective termination of one twin for severe twin-twin transfusion syndrome at 20 + 4 gestational weeks; her coagulation parameters resolved postpartum^38^.

In the United States, DIC accounts for up to 25% of maternal deaths^39^ and increased by 83% during the postpartum hospitalization between 1998 and 2009^40^. Although the most common obstetrical cause for DIC in pregnant women is considered massive obstetric hemorrhage^41–43^, the most common cause for DIC in pregnant women suffering IUFD is acute extensive placental abruption with an incidence of 0.5% to 1%^3,44^. In total, placental abruption accounted for 1.1% of direct maternal perinatal mortality in the United States between 2006 and 2010^39^. Common features of a non-concealed acute placental abruption are vaginal bleeding, severe abdominal pain, uterine tenderness or tetanic contractions^45^. The acute separation of the implanted placenta from the decidua causes large amounts of decidual and trophoblast-derived tissue factors to enter the maternal circulation and activate profound coagulation^46^. Rapid treatment of the underlying disorder is considered the cornerstone of DIC management^47^. While the release of fetal tissue factors during maceration may well be associated with a local reaction of the surrounding environment, it is plausible to assume that intact amniotic membranes and the constriction of spiral arteries act as a natural barrier and inhibit further feto-maternal shift of endotoxins. This may prevent the crossing over of potential mediators for systemic inflammation or DIC and natural waste products after IUFD.

The strengths of this study are, firstly, its strict inclusion and exclusion criteria, resulting in a homogenous cohort limited to singleton IUFDs and devoid of any obvious maternal or fetal risk factors for DIC, apart from the fetal maceration grade, which was subject to our hypothesis. Secondly, we applied the Erez score instead of the International Society for Thrombosis and Hemostasis (ISTH) scoring system for DIC^47^, since evaluation of D-Dimer has not been routinely assessed in this cohort due to its poor positive predictive value in pregnancy^48,49^. The Erez score, however, puts emphasis on the PC count, and thrombocytopenia has been appreciated as one of the commonest diagnostic features for DIC^50^. Last but not least, the single-center setting of this study ascertained a continuous degree of quality in laboratory assessment and reporting of autopsy examinations, restricting the influence of bias and heterogeneity in reporting across centers. Despite these strengths, we acknowledge certain study limitations. Due to its retrospective study design, we were unable to control for accuracy in documentation of medical co-morbidities, pre-existing coagulopathies and concomitant anticoagulation therapy, as this was self-reported by the pregnant woman and therefore subject to recall bias at time of antenatal booking. Furthermore, inherent to its study setting, the total number of our population was relatively small, especially for women who experienced DIC without other common risk factors, as outlined in the methods section. Whilst in our database 37.4% of fetuses were diagnosed with a maceration grade of III (≥8 days), the exact percentage of women whose baby had died more than 28 days ago, is unclear. The longer the fetus remains dead in utero, however, the higher the likelihood of developing DIC, as described in previous studies^51,52^. Owing to the tight antenatal follow-up regime at our institution, however, the majority of IUFD would have been timely recognized within at least 6 weeks of the last viability check. Furthermore, we acknowledge the fact that DIC is a dynamic process and that women might have still developed DIC upon their discharge from hospital, which we have not taken into account in this study due to lacking follow-up data. These limitations should prompt further validation of our findings in larger prospective multi-center cohort studies as well as future research on the exact pathophysiology of DIC and its timeline after fetal death.

In conclusion, our study shows that the occurrence of DIC in women after singleton IUFD is independent of the fetal maceration grade. We herewith support the clinical value of the Erez score in identifying women at risk of DIC, independent of the precipitating factor. Of note, our work is of theoretical value, challenging a hypothesis that has been established over a century ago, and which has only been backed up by a sparse body of evidence.

## Methods

### Study design and data collection

We conducted a retrospective cohort study on women having delivered a stillborn fetus between February 2003 and February 2017 at the Department of Obstetrics and Fetomaternal Medicine at the Medical University of Vienna, Austria. We retrieved all delivery entries from the local obstetrical database ViewPoint® (Version 5.6.16.917; General Electric Company, Wessling; Germany) using the criteria (a) singleton pregnancy, (b) Apgar score of 0 at 1, 5 and 10 minutes, (c) ticked check-box for “stillbirth”. All collected data were manually reviewed for accuracy and compliance with our inclusion and exclusion criteria. Inclusion criteria were singleton IUFD above gestational week 22 + 0, documentation of the fetal maceration grade following conventional post-mortem autopsy and availability of maternal coagulation parameters (platelet count, prothrombin time and fibrinogen) at the time of IUFD-diagnosis and/or upon hospital-admission and up to 2 days after delivery. Exclusion criteria were multiple pregnancies, IUFD following selective embryo reduction, medical or surgical terminations of pregnancies, perinatal fetal deaths and all independent risk-factors that are known to cause DIC in pregnant women, including acute postpartum hemorrhage due to uterine atony, trauma or retained tissue; placental abruption; severe preeclampsia; HELLP; maternal sepsis or intra-amniotic infection; acute fatty liver and amniotic fluid embolism. Following those search criteria, we retrieved a total of 612 cases from the ViewPoint® database, which we further limited to 193 cases after considering the above inclusion and exclusion criteria. Among those, 75 women had missing blood tests and 27 fetuses had not undergone post-mortem autopsy. Hence, our final study-cohort constituted of 91 eligible cases (s. Supplementary Fig. 1).

### Definitions

Maternal age was defined as age in years at the time of delivery; gravidity was defined as the number of the current pregnancy; parity was defined as the number of previous deliveries. Ethnicity, nicotine, alcohol and illicit drug consumption were self-reported by the woman at the time of antenatal booking. Body mass index (BMI) at booking was grouped as obese (≥30 kg/m^2^), overweight (25–29 kg/m^2^), normal (19–24.9 kg/m^2^) and underweight (<18.9 kg/m^2^). Cause of death was defined as the “initial, demonstrable pathophysiological entity initiating the chain of events that has irreversibly led to death” and was categorized according to the Tulip classification upon collection of all relevant maternal, fetal, obstetrical and post-mortem information^53^.

Postpartum hemorrhage (PPH) was differentiated into minor PPH as blood loss between 500–1000 ml within the first 24 hours of delivery and major PPH defined as blood loss exceeding 1000 ml^54^. DIC was diagnosed based upon abnormal global hemostatic tests, as reflected by an Erez score ≥26, up to five days after delivery, presence of minor to major PPH and/or critical signs of beginning or fulminant organ failure. Women with DIC were assigned an ICD-10 code [D65-D69] in their medical records.

### Maceration grade

All fetal autopsies were conducted by an expert team of three perinatal pathologists according to standardized guidelines at the Clinical Institute for Pathology, Medical University of Vienna, Austria. Maceration grading was based upon external fetal features and allowed estimation of time relapsed since intrauterine death. Maceration grade 0 referred to “parboiled” skin discoloration (<8 hours of death); maceration grade I described not specified desquamation of the skin (≥8 hours); maceration grade II was assigned to fetuses with blood stained effusions in serous cavities and skin peeling (2–7 days), and maceration grade III corresponded with the appearance of a yellow-brown liver, turbid effusions and mummification (≥8 days)^20^. Fetal autopsy reports were derived from the local electronic hospital database. Reports with missing data were excluded.

### Placenta histology

Placental histology was carried out by placental histopathologists at the Clinical Institute for Pathology, Medical University of Vienna, Austria. Placental lesions were reported according to local guidelines based upon the Amsterdam Placental Workshop Group Consensus Statement^55^. Placental lesions were then classified into nine categories as per Turowski *et al*. (1. Normal placenta according to gestational age; 2. Chorioamnionitis; 3. Villitis and intervillositis; 4. Maternal circulatory disorders (decidual vasculopathy); 5. Fetal circulatory disorders; 6. Delayed villous maturation; 7. Suggestive for genetic aberration; 8. Implantation disorders; 9. Other lesions)^56^.

### DIC - Scoring system

The Erez score is a pregnancy-adapted scoring system to early identify women at risk for DIC^26^. In contrast to the International Society for Thrombosis and Hemostasis (ISTH) scoring system for DIC^47^, the Erez score is omitting the consideration of fibrin degradation products (D-Dimer) which can yield a false-positive result in pregnancy. Instead, it is the sum of the platelet count (PC), prothrombin time (PT; a ratio between the patient’s value and the inferior reference value, both given in seconds) and fibrinogen (Fib): [**PC** > 185 × 109/l = 0 OR PC (100 × 109/l) − (185 × 109/l) = 1 OR PC (50 × 109/l) − (100 × 109/l) = 2 OR PC < 50 × 109/l = 1] + [**PT** < 0.5 = 0 OR PT 0.5–1 = 5 OR PT 1.0–1.5 = 12 OR PT > 1.5 = 25] + [**Fib** 3.0 g/l = 25 OR Fib 3.0–4.0 g/l = 6 OR Fib 4.0–4.5 g/l = 1 OR Fib > 4.5 g/l = 0]. An Erez score of ≥26 suggests high probability for DIC.

### Laboratory testing

Biochemical analysis was conducted at the Department of Laboratory Medicine, Medical University of Vienna, Austria (ISO9001-certification; ISO15189-accreditation). Platelet count (PC) was quantified on Sysmex hematology analyzers (Sysmex Europe GmbH, Norderstedt, Germany). Activated partial thromboplastin time (aPTT), thrombin time (TT) and fibrinogen according to Clauss was measured on STA-R analyzers with CE-certified assays (Diagnostica Stago, Asniéres, France). Prothrombin times (PT) were assessed according to Owren (until 2015-08-18 Normotest® CE [Technoclone GmbH, Vienna, Austria]; thereupon Hepato-Prest CE [Diagnostica Stago]) on STA-R analyzers (Diagnostica Stago). For calculations, results below the limit of detection (LOD) were estimated by their assumed expected value: $\bar{x}=\frac{0+LOD}{2}$ and values above the highest point of the standard curve (HQS) were fixed at: ${x}_{h}=HQS+1$. At our institution, normal ranges for PC are 150–350 G/L, for PT 75–140% and for Fib 2.0–4.0 g/dl.

Maternal laboratory test results were extracted from the Laboratory Information and Management System MOLIS (vision4health, Bochum, Germany) at the Department of Laboratory Medicine. As DIC is known to be a dynamic process, we retrieved the lab reports for each subject at different stages throughout the treatment period, namely at the date of IUFD-diagnosis, date of hospital admission and up to 5 days after delivery. In case of dynamic changes in lab results, the poorest values have been evaluated in this study for each parameter. Variables were checked and validated for completeness and accuracy by the study investigators.

### Statistical analysis

Continuous data is presented as mean (±standard deviation, SD), if normally-distributed, otherwise as median (range). Categorical data is given as counts and percentages. Relationships between continuous variables were assessed by Spearman’s rank correlation tests. Differences in continuous physiological parameters and laboratory results were calculated using the Kruskal-Wallis test and ANOVA, respectively. All reported *p*-values are two-sided, and a Greenhouse-Geisser corrected *p*-value was considered as level of significance (*p* < 0.05). Statistical tests were performed with SPSS® version 22.0.0 (IBM, Armonk, NY, USA) and GraphPad Prism® 7.0c (GraphPad Software Inc., La Jolla, CA, USA). Figures were compiled by GraphPad Prism® 7.0c.

The study was approved by the Ethics Committee of the Medical University of Vienna (EK 1231/2017) and complied with the principles outlined in the declaration of Helsinki. Patients’ written consent was not required as per *Austrian Federal Act concerning Protection of Personal Data* (DSG 2000).

## Electronic supplementary material

Supplementary information

## Data Availability

The datasets generated and analyzed during this study are available from the corresponding author on reasonable request.
