# Supplementary material for: Impact of fetal maceration grade on risk of maternal disseminated intravascular coagulation after intrauterine fetal death – A retrospective cohort study
Source: Sci Rep. 2018 Aug 24;8:12742. doi: 10.1038/s41598-018-30687-0 (PMC6109103; doi:10.1038/s41598-018-30687-0)
Supplement: Supplementary file 1 — Supplementary information [file 41598_2018_30687_MOESM1_ESM.pdf]

Impact of fetal maceration grade on risk of maternal disseminated intravascular coagulation after intrauterine fetal death – A retrospective cohort study

Dana A. Muin[1,2], Helmuth Haslacher[3], Vanessa Koller[1], Herbert Kiss[1*], Anke Scharrer[4], Alex Farr[1]

[1] Department of Obstetrics and Gynecology, Division of Fetomaternal Medicine, Medical University of Vienna, 1090, Vienna, Austria

[2] Tommy's Stillbirth Research Center, Faculty of Biology, Medicine and Health, The University of Manchester, Manchester, M13 9WL, United Kingdom

[3] Department of Laboratory Medicine, Medical University of Vienna, 1090, Vienna, Austria

[4] Clinical Institute for Pathology, Medical University of Vienna, 1090, Vienna, Austria

***Corresponding Author:**

Herbert Kiss, MD, MBA

Medical University of Vienna

Department of Obstetrics and Gynecology, Division of Fetomaternal Medicine

Waehringer Guertel 18–20, 1090 Vienna, Austria

Tel: +43 1 40400 28220, *Fax:* +43 1 40400 28620

Email: herbert.kiss@meduniwien.ac.at

Supplementary Figure 1 Flow diagram on the patients' selection from ViewPoint® database between 2003–2017

Abbreviations: DIC Disseminated intravascular coagulation; *n* Number; † Acute postpartum hemorrhage (secondary to uterine atony, trauma or retained tissue), placental abruption, severe pre-eclampsia, HELLP, maternal sepsis, intra-amniotic infection, acute fatty liver, amniotic fluid embolism.

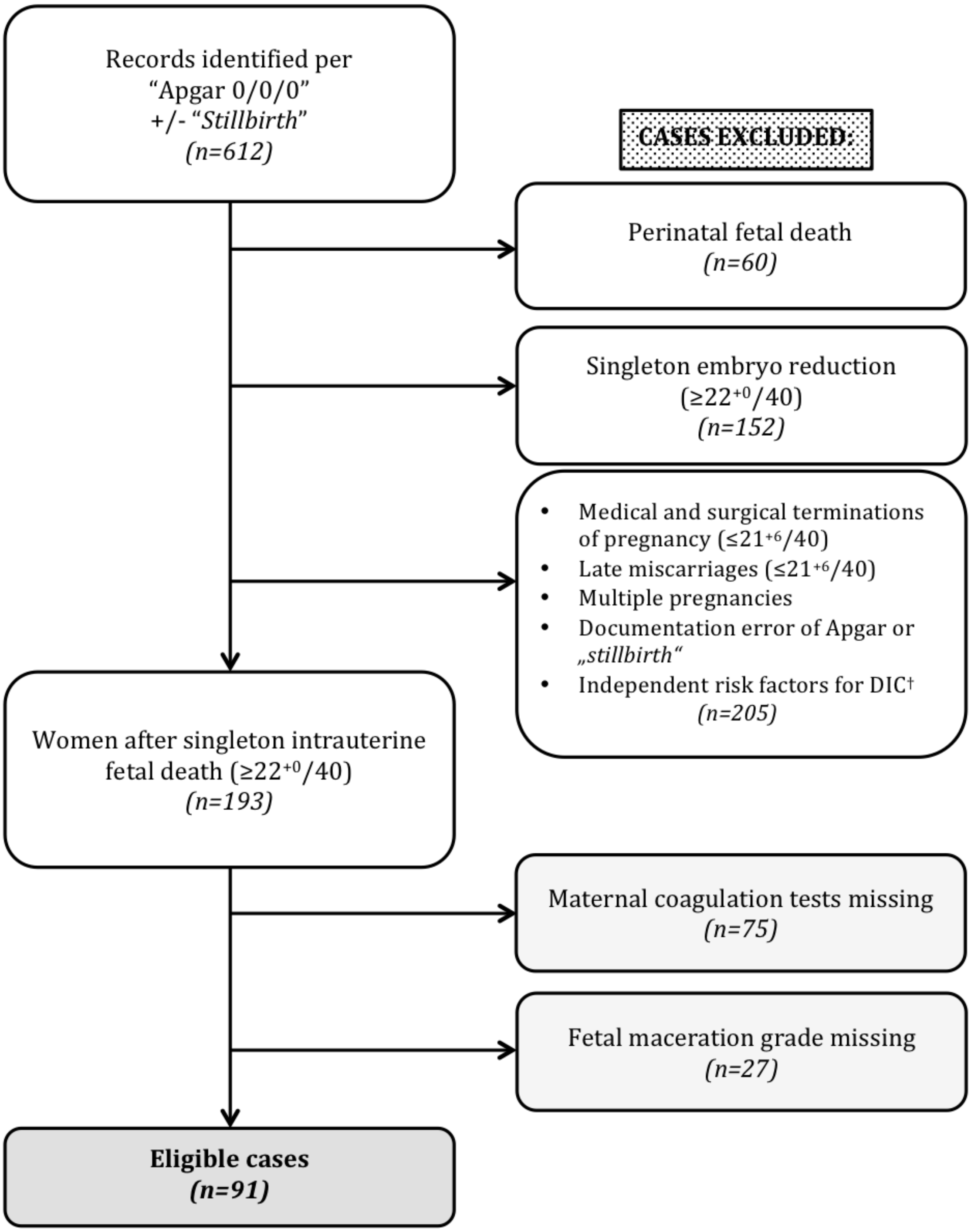

Supplementary Table 1 Maternal characteristics of women with positive Erez score (n=6) *Abbreviations: IUFD* Intrauterine fetal death; *IUGR* Intrauterine growth restriction; *LSCS* Lower segment caesarean section; *n/a* not applicable due to missing data

	Patients Nr	1	2	3	4	5	6
Maternal characteristics	**Ethnicity**	Middle European, white Caucasian	Middle European, white Caucasian	Eastern European, white Caucasian	Middle European, white Caucasian	Middle European, white Caucasian	Middle European, white Caucasian
	**Maternal age [years]**	39	31	25	33	35	33
	**BMI [kg/m2]**	n/a	n/a	n/a	n/a	26.35	32.95
	**Gravida**	1	3	1	1	2	2
	**Para**	0	0	0	0	1	1
	**Patient's history**	Unremarkable	2x previous miscarriages	Unremarkable	Lupus erythematodes	1x previous LSCS	Unremarkable
	**Mode of conception**	In-vitro fertilisation	Spontaneous	n/a	Spontaneous	Spontaneous	Spontaneous
	**Nicotine**	no	no	n/a	no	no	no
	**Alcohol**	no	no	no	no	no	no
	**Illicit drugs**	no	no	no	no	no	no
IUFD-Pregnancy characteristics	**Gestational weeks (at IUFD)**	25	22	23	24	38	39
	**(+) Gestational days**	3	6	6	5	1	0
	**Fetal sex**	Female	Male	Female	Male	Male	Female
	**Pathologies during pregnancy**	IUGR, Oligohydramnios	IUGR	None	IUGR, treated pre-eclampsia	IUGR	Insulin-treated gestational diabetes, polyhydramnios
Pathology report	**Fetal pathology (as per autopsy report)**	Massive cardiac hypertrophy	Bilateral cleft lip, subcapsular liver hematomas, fresh brain haemorrhage	Cardiac hypertrophy	None	None	None
	**Maceration Grade**	0	0	I	II	II	III
	**Fetal weight [g]**	500	365	600	500	2560	3700
	**Fetal growth centiles**	0.3	0.3	47.8	0.4	2.5	63.9
	**Cord pathology**	None	None	Edematous Wharton's jelly	None	2x umbilical cord entanglement, long umbilical cord (64 cm)	True umbilical knot, long umbilical cord (≥40 cm)
	**Placental histology (Turowski et al.)[53]**	Maternal circulatory disorder (Category 4); Delayed villous maturation (Category 6)	Maternal circulatory disorder (Category 4)	Normal (Category 1)	Maternal circulatory disorder (Category 4)	Implantation disorder (Category 8)	Normal (Category 1)
	**Cause of IUFD (Tulip)**	2.2.1. Placental bed pathology + 2.2.2. Placental development pathology	1.11.2. Congenital anomaly - other (multiple organs)	6. Unknown - Despite thorough investigation	2.2.1. Placental bed pathology + 2.2.2. Placental development pathology	2.3. Umbilical cord complication	2.3. Umbilical cord complication
Coagulation parameters	**Blood loss [ml]**	500	150	800	200	500	100
	**Platelet count [G/L]**	97	46	29	108	67	62
	**Prothrombin time [%]**	104	78	25	75	59	68
	**Fibrinogen [g/dl]**	2.7	2.66	1.93	2.85	1.93	1.14
	**Erez Score [pos ≥27]**	27	26	51	26	52	39
